# Representations of machine vision technologies in artworks, games and narratives: A dataset

**DOI:** 10.1016/j.dib.2022.108319

**Published:** 2022-05-28

**Authors:** Jill Walker Rettberg, Linda Kronman, Ragnhild Solberg, Marianne Gunderson, Stein Magne Bjørklund, Linn Heidi Stokkedal, Kurdin Jacob, Gabriele de Seta, Annette Markham

**Affiliations:** a University of Bergen, Norway; b Royal Melbourne Institute of Technology, Australia

**Keywords:** Machine vision, Algorithmic culture, Digital Humanities, Science Fiction, Digital art, Video games, Narrative, Media studies, Literary studies, Facial recognition, DH, Digital Humanities, Sci-fi, Science fiction

## Abstract

This data paper documents a dataset that captures cultural attitudes towards machine vision technologies as they are expressed in art, games and narratives. The dataset includes records of 500 creative works (including 77 digital games, 190 digital artworks and 233 movies, novels and other narratives) that use or represent machine vision technologies like facial recognition, deepfakes, and augmented reality. The dataset is divided into three main tables, relating to the works, to specific situations in each work involving machine vision technologies, and to the characters that interact with the technologies. Data about each work include title, author, year and country of publication; types of machine vision technologies featured; topics the work addresses, and sentiments shown towards machine vision in the work. In the various works we identified 874 specific situations where machine vision is central. The dataset includes detailed data about each of these situations that describes the actions of human and non-human agents, including machine vision technologies. The dataset is the product of a digital humanities project and can be also viewed as a database at http://machine-vision.no. Data was collected by a team of topic experts who followed an analytical model developed to explore relationships between humans and technologies, inspired by posthumanist and feminist new materialist theories. The dataset is particularly useful for humanities and social science scholars interested in the relationship between technology and culture, and by designers, artists, and scientists developing machine vision technologies.

## Specifications Table

**Table:** 

Subject	Humanities (General)
Specific subject area	Digital Humanities, Digital Culture, Art History, Game Studies, Media Studies, Literary Studies, Science and Technology Studies, Visual Studies
Type of data	Text, table.
How data were acquired	Data were generated by a team of topic experts who qualitatively selected and analysed relevant artworks, games and narratives (e.g. movies and novels). An analytical model was developed to describe relationships between technologies and human and nonhuman agents in the artworks, games and narratives, and the model was encoded in a Drupal database. Descriptive and interpretative data about each work was identified and logged in the database according to the analytical model. Data were then exported from the database in csv format.
Data format	Raw, Filtered
Description of data collection	The project identified relevant works by searching databases, visiting exhibitions and conferences, reading scholarship, and consulting other experts. The inclusion criteria were creative works (art, games, narratives) where one of the machine vision technologies listed in Table 2: Table 3 was used in or represented by the work. The collection happened between January 2019 and September 2021.
Data source location	The primary data sources are the actual creative works, which are not included in this dataset. The dataset consists of secondary data documenting and analysing the primary data. A complete list of the primary data sources can be found in creativeworks.csv. Narratives include: • Published novels and short stories • Movies and TV series screened at cinemas or film festivals and available on public broadcasting or commercial streaming services • Written narratives, such as fan fiction, creepypasta, short stories and electronic literature published in online journals, websites or public forums • Electronic literature published online or presented in public exhibitions • Music videos with strong narrative elements Games include: • Video games available for purchase or download in stores or on platforms such as Steam Artworks include: • Artworks publicly displayed in exhibitions or online
Data accessibility	Repository name: UiB Open Research Data / DataverseNO Data identification number: doi: 10.18710/2G0XKN Direct URL to data: 10.18710/2G0XKN
	Citation for dataset: Rettberg, Jill Walker; Kronman, Linda; Solberg, Ragnhild; Gunderson, Marianne; Bjørklund, Stein Magne; Stokkedal, Linn Heidi; de Seta, Gabriele; Jacob, Kurdin; Markham, Annette, 2022, "A Dataset Documenting Representations of Machine Vision Technologies in Artworks, Games and Narratives", 10.18710/2G0XKN, DataverseNO, V1 The database that the dataset was exported from can be viewed at http://machine-vision.no. A permanent, static archive of the database is available as a set of HTML and CSS files that can be cited as follows: Rettberg, J. W., Kronman, L., Solberg, R., Gunderson, M., Bjørklund, S. M., Stokkedal, L. H., de Seta, G., Jacob, K., & Markham, A. (2022). Database of Machine Vision in Art, Games and Narratives: Archival Version in HTML and CSS (Version 1.0.0) [archived database]. URL: https://machinevisionuib.github.io10.5281/zenodo.6514729 In addition to the R code included with the dataset itself and described in this paper, the R code required to generate the figures in this data paper is available on Github: Rettberg, J. W. (2022). Scripts for analysing data from 'A Dataset Documenting Representations of Machine Vision Technologies in Artworks, Games and Narratives' (Version 1.0.1) [Computer software]. 10.5281/zenodo.6510181

## Value of the Data

• The dataset documents how machine vision technologies are imagined in the highly influential cultural discourses of narratives, art, and games.
• These data are primarily useful to scholars in disciplines such as Digital Culture, Digital Humanities, Science and Technology Studies, Literary Studies, Media Studies, Game Studies and Visual Studies. They could also be useful for designers, artists and scientists developing machine vision technologies.
• The data can be reused for humanities and social science-based research on machine vision, and to compare and contrast representations of technologies versus actual development and widespread use of technologies.
• The data combines interpretative and descriptive categories and focuses on features, characteristics and actions that can generate further research questions.
• The analytical model and database structure used to capture cultural representations of technology may be useful for other projects that seek to register and analyse large amounts of cultural data that cannot be easily segmented into clearly defined categories.

## 1 Data Description

The dataset includes data describing 77 games, 190 artworks and 233 narratives (in total 500 Creative Works) where machine vision technologies play an important role. The data are qualitative analyses by the research team, and were not automatically scraped or extracted from the Creative Works. The dataset that is described in this data paper can be downloaded from DataverseNO [1]. A static archive of the database the dataset was exported is also available as a set of HTML and CSS files that can be viewed in a web browser [2].

**Fig. 1:**
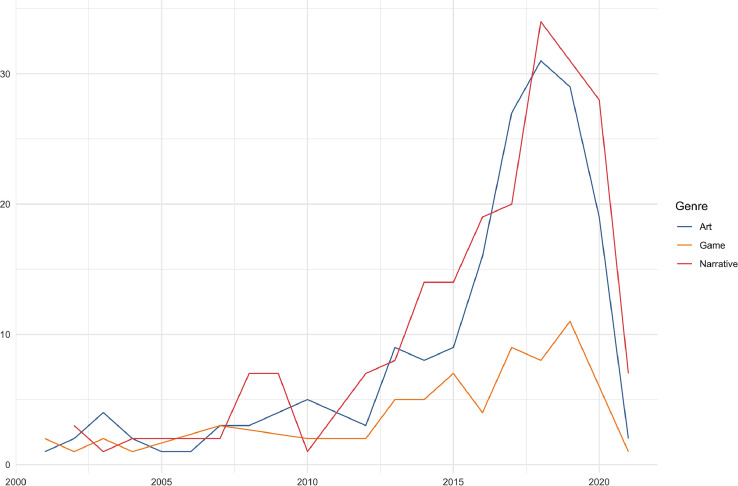
Year of publication for creative works. The 34 works published before 2000 are not included in this figure. The R code used to generate this and the other figures in this paper is available at Github [3].

80% of the Works are from 2011 to 2021, and just over half from 2016 to 2021. 34 works were published between 1891 and 1999. Fig. 1 shows the distribution of the 464 works published in 2000 or later by year.

**Fig. 2:**
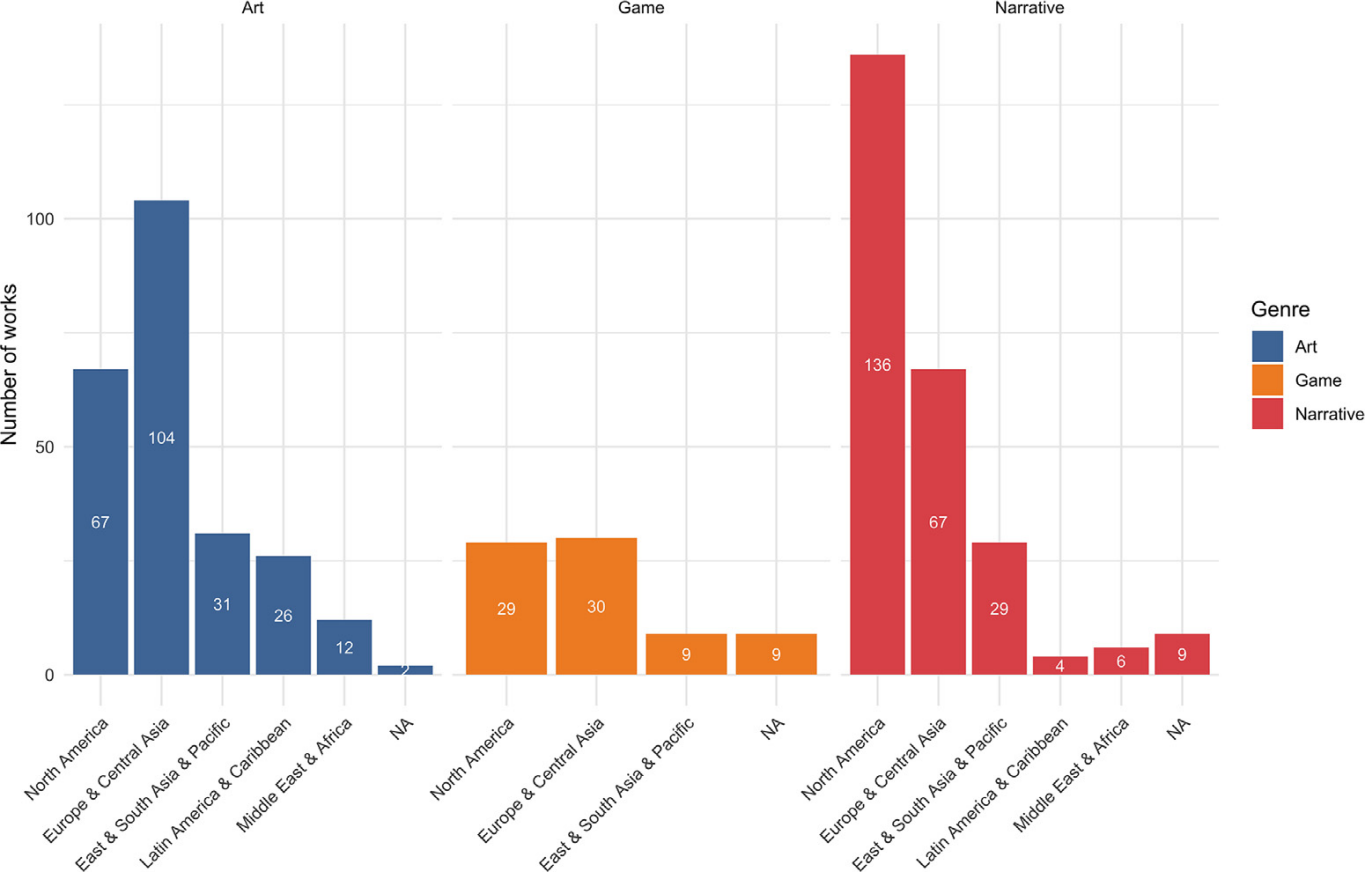
Geographic distribution of creative works. Note that some works are affiliated with more than one country, so the total is more than 500. Regions are grouped by the seven regions of the World Bank's Economic Indicators. NA refers to works lacking information about an affiliated country.

The Creative Works are from 59 different countries, with 78,6% from North America and Europe, and 21,4% from other parts of the world. As shown in Fig. 2, narratives (especially movies, novels and TV-series) are more heavily biased towards English-speaking countries, while artworks and games are more evenly spread. Attempts to mitigate the bias are described in the discussion of the selection process below.

The three main files contain information about Creative Works, Situations, Characters (see Table 1). The data are exported from a relational database (http://machine-vision.no) we developed for the project, as described in the methodology section below.

**Table 1:** Files included in the dataset. 00_README.txt is a plain text file, and machinevisionscripts.R is a text file containing scripts that can be run in the programming language R. All other files are csv files encoded in UTF-8.

Filename	Content	Data structure
00_README.txt	Plain text description of the dataset.	Plain text. Describes the files and includes a list of all 500 works in the dataset.
01_codebook.csv	Lists variables used in the different data files with definitions and information about which variables are included in each file.	Variable, Description, PossibleValues, MultiplesPossible, and a column for each of the other files in the dataset indicating whether a variable is included in the file.
02_technologies_sentiments _topics_definitions.csv	Lists definitions of each of the technologies, sentiments and topics.	Term, Type, Definition
creativeworks.csv	Lists data describing each of the 500 Creative Works in the dataset.	WorkID, WorkTitle, Year, Country, Genre, TechRef, TechUsed, Topic, Sentiment, Situation, SituationID, Character, CharacterID
situations.csv	Lists situations involving machine vision technologies in the Creative Works with details about the actions of humans, technologies, and other agents.	SituationID, Situation, Genre, Character, Entity, Technology, Verb
characters.csv	Lists all Characters that interact with machine vision with fields describing how they are represented in the Creative Work.	CharacterID, Character, Species, Gender, RaceOrEthnicity, Age, Sexuality, IsGroup, IsCustomizable
narrativegenres.csv	Lists genres of Creative Works that have been classified as Narratives.	WorkID, WorkTitle, Genre (Movie, Novel, Short story, TV series or episode, Fan fiction, Creepypasta, Comic, Electronic Literature, Music Video, Online Video)
situation_description.csv	Written descriptions of each situation and quotations from texts where available.	SituationID, Situation, SituationDescription, SituationQuotes
situations_visual.csv	Lists colours and aesthetic characteristics of situations, when relevant. Notes whether situation is shown from the machine's point of view.	SituationID, Situation, Colours, AestheticCharacteristics, MachinePOV
creators.csv	Lists all creators (authors, artists, producers) of works with Wikidata IDs where available.	Creator, Creator_WikidataID
worksinfo.csv	Bibliographic data about each creative work, including Wikidata Q IDs when available to enable interoperability.	WorkID, Variable and Value. Variables include WorkTitle, Year, Genre, Creator, URL, Country, Work_WikidataID, IsSciFi.
machinevisionscripts.R	Scripts to load data files with levels in R, to join data files, merge categories, create contingency tables that show the count of occurrences instead of a list of each occurrence, and transform worksinfo.csv to a wide, more human-friendly table.	R scripts. These can be run in R or RStudio or viewed in a text editor.

The data are represented in a two-dimensional comma separated table with a separate row for each unique combination of values. Since the *creativeworks.csv* and *situations.csv* files have multiple values for several variables, with a separate row for each combination of values, care must be taken to remove duplicate values before doing any frequency analysis. See Tables 3 and 4 and the accompanying explanation for more details on the formats.

In the following, we describe the three main categories of data: creative works, situations and characters and how they relate to each other. We then describe the methodology in the next section.

### 1.1 Machine Vision Technologies

Before describing the files it is useful to understand which technologies the dataset focuses on. Based on our initial survey of Creative Works, we selected 26 technologies that were commonly referenced or used.

Table 2 lists the technologies with the definitions we used when coding the data. The definitions are cultural definitions developed by the project team to align with the way the technologies are portrayed in Creative Works and are not intended to be technically precise.

**Table 2:** Definitions of the Machine Vision technologies.

Technology	Definition
3D scans	3D images or models produced by surface scanning such as photogrammetry, LiDAR, 3D scanning, etc. Does not usually include holograms unless the surface scan that produced the hologram is presented as particularly important in the game.
AI	General purpose artificial intelligence systems that can perform a broad range of intellectual and cognitive tasks. A system that can only perform one task, such as facial recognition, video recommendations, conversations with humans, or playing a game, is not tagged as AI but as machine learning, if central to the work.
Augmented reality	Digital images overlay or appear to be integrated into a physical environment. Unlike holograms, glasses, implants or screens are usually required to view AR.
Biometrics	Technologies used to identify an individual. Can include retina scans, gait recognition, DNA phenotyping, fingerprint scans. Facial recognition is not included here but tagged separately.
Body scans	Any imaging technology that shows parts of the body that are usually hidden, for instance brain imaging, fMRI, ultrasound, x-ray, or body scans in airport security that show objects under clothing. Includes cameras probes that enter the body.
Camera	Single-purpose, portable camera technologies for creating a visual representation, e.g., camcorders, SLR cameras, cinema cameras. Does not include CCTV, satellites or cameras that are part of other technologies, such as webcams and camera phones, and does not include cameras that produce non-visual information such as motion tracking.
Cameraphone	Cameras integrated into phones that are easily carried on the person and often include computational capabilities.
Deepfake	Technologies that use machine learning to generate videos that strongly resemble a specific human being. For our purposes, we include for instance art, satire and spoofs, but not professional uses of synthetic or virtual actors in Hollywood movies.
Drones	Remote controlled or autonomous aerial vehicle with a camera. Does not carry a human operator.
Emotion recognition	Software that analyses facial expressions to infer a person's emotions and inner states of mind.
Facial recognition	Automated identification of identity based on a face. This could mean identifying a face as belonging to a specific individual, or to a gender, race or other category.
Filtering	Applying filters to an image to enhance or remove certain aspects, e.g., Instagram filters, beautifying filters, selfie lenses.
Hologram	A 3D projection of archived material or real-time events. It usually features humans, nonhumans or terrain and can usually be seen without special glasses, screens or implants.
Image generation	Synthetic images generated with the use of technologies such as GANs and other neural networks. Does not include animation in general.
Interactive panoramas	360 images of the real world that are stitched together to allow movement between photographed spots. Includes various street view services.
Machine learning	The capacity of a computer to learn from experience, i.e. to modify its processing based on newly acquired information. Includes neural networks and machine learning datasets.
Microscope/Telescope	Any kind of technology that allows us to see objects that are too small or too far away to be clearly viewed with the naked human eye. Can include rifle scopes if the zoom is quite strong.
Motion tracking	Technologies that register movements. Does not include GPS locations and other remote tracking.
Non-visible spectrum	Technologies designed to register objects, shapes and movements in low light conditions, often enhancing the spectral range, e.g. using night vision, infrared, near infrared and ultraviolet. Sources of light such as torches are not included.
Object recognition	Automated identification of an object using visual data. For our purposes, we do not include facial recognition, which has a separate tag. A fingerprint would be tagged with "Biometrics", but not "Object Recognition", despite the purely technical level being similar.
Ocular implant	An implant of some sort has been inserted into somebody's eyes or brain, usually providing enhanced vision, AR displays or recordings of all that is seen.
Satellite images	Images collected by a satellite. Includes Google Earth and many others.
Surveillance camera	CCTV, IP cameras, home surveillance cameras, baby monitors.
UGV	Remote controlled or autonomous ground vehicle with a camera. Does not carry a human operator. Can include autonomous vehicles and drones that work underground or underwater.
Virtual reality	Immersive experiences taking place in computer-generated simulated realities, usually involving headsets or similar equipment.
Webcams	Video cameras connected to personal computers that stream images in real time to the internet.

### 1.2 Creative Works

The first level of analysis is that of the works, that is of the novels, movies, videogames, artworks and so on in our sample. *Creativeworks.csv* includes four data types about each of the 500 Creative Works: provenance, content description, sentiment, and identification of situation. *Provenance* includes the fields Title, Year, Genre, and country of origin. *Content description* includes machine vision technologies referenced (TechRef), machine technologies used (TechUsed), topics (subject matter) and characters who interact with machine vision technologies in the work. The identification of sentiment, which is qualitative and interpretive rather than descriptive or computationally inferred data, encapsulates attitudes towards machine vision technologies as they are represented in the Work. Situations identify selective situations within Works that involve machine vision technologies and demonstrate how agency is distributed between human and non-human actors (see Table 6: Fields in the file situations.csv. Table 6 for more details).

We have interpreted a “work” broadly, counting a whole TV series like *Black Mirror* as a single work and in some cases combining both a series of novels and the movie adaptations as a single work, as with *The Hunger Games*.

Several of the variables can have multiple values, as shown in Table 3, which presents just the TechRef and Sentiment variables for two creative works. For clarity, we have exported the data with a separate row for each unique combination of values, as shown in simplified form in Table 4.

**Table 3:** A simplified table showing that a single Creative Work may have multiple values for some fields.

WorkTitle	TechRef	Sentiment
Minority Report	Augmented Reality, Biometrics, Facial Recognition	Hostile, Intrusive, Oppressive
Horizon Zero Dawn	AI, Augmented Reality, Biometrics, Holograms	Alien, Helpful, Intimate

**Table 4:** The file creativeworks.csv presents the data with a separate row for each unique set of values. This is a simplified extract showing how the Technologies Referenced and Sentiments are represented in the file.

WorkTitle	TechRef	Sentiment
Minority Report	Augmented Reality	Hostile
Minority Report	Biometrics	Hostile
Minority Report	Facial Recognition	Hostile
Minority Report	Augmented Reality	Intrusive
Minority Report	Biometrics	Intrusive
Minority Report	Facial Recognition	Intrusive
Minority Report	Augmented Reality	Oppressive
Minority Report	Biometrics	Oppressive
Minority Report	Facial Recognition	Oppressive
Horizon Zero Dawn	AI	Alien
Horizon Zero Dawn	Augmented Reality	Alien

**Table 5:** Descriptions of the fields in creativeworks.csv.

Column header	Description	Multiple values possible
WorkID	A number that uniquely identifies the Creative Work in the dataset.	No
WorkTitle	The title of the work. Series and adaptations (e.g., novels made into movies) are logged as a single entry unless machine vision is presented very differently in the different episodes or versions.	No
Year	The year of publication. In the case of series and adaptations, the year when the first episode or version was published or released.	No
Genre	The genre that the Creative Work belongs to (Art, Game or Narrative).	No
Country	The main country or countries the creators of the work are affiliated with, for instance by citizenship or residency.	Yes
TechRef	Machine vision technologies described, represented or mentioned in the Creative Work. See Table 2 for a list of all 26 Technologies.	Yes
TechUsed	Machine vision technologies used by the Creative Work. Examples might include an artwork that uses facial recognition of museum visitors to generate a customised output, or a game that uses eye-tracking so that the player can interact with the game by blinking or moving their eyes. See Table 2 for a list of all 26 Technologies.	Yes
Topics	Topics that categorise the various Creative Works based on their explicit subject matters. A Topic is foregrounded and central in the Creative Work, with one Creative Work usually having up to five Topics. Topics are tagged with one or more of the following words: AI, Animals, Automation, Autonomous vehicles, City, Climate change, Companionship, Competition, Conflict, Consciousness, Crime, Cyborg, Dystopian, Economy, Empathy, Family, Free will, Gender, Grief, Hacking, Horror, Identity, Inequality, Labour, Nature, Nudity, Physical violence, Playful, Race and ethnicity, Robot/android, Romantic relationship, Sex, Social media, Surveillance, Utopian, War.	Yes
Sentiment	The attitudes towards the machine vision technology represented in the Creative Work. Sentiments are tagged with one or more of the following words: Alien, Creepy, Dangerous, Disgusting, Empowering, Exciting, Flawed, Fun, Helpful, Hostile, Intimate, Intrusive, Misleading, Neutral, Oppressive, Overwhelming, Prosocial, Protective, Subversive, Wondrous.	Yes
Situation	The titles of Machine Vision Situations in this Creative Work. A Situation is a specific scene or event that involves machine vision technologies.	Yes
SituationID	A number that uniquely identifies the situation in the dataset.	Yes
Character	The names of characters in the work who engage with machine vision technologies.	Yes
CharacterID	A number that uniquely identifies the character in the dataset.	Yes

This format makes it easy to create adjacency tables for network analysis between two values, or to select just the needed variables (columns) and values. However, it is important to realise that the format means that values are repeated.

Doing a frequency analysis on *creativeworks.csv* without filtering out duplicates will thus lead to vastly inflated and incorrect results. If you want to identify how common Facial Recognition is in movies, for instance, you need to filter out duplicate rows. As you can see in Table 4, Facial Recognition is listed three times for *Minority Report*. In the full dataset *creativeworks.csv* there are 442 rows just for *Minority Report*, and 72 rows include the value Facial Recognition. A work with more characters or situations will have many more rows than a work with only one situation and one character, because there will be so many more unique combinations of values. The TV series *Black Mirror* has the most rows in the dataset (26,880), while four works only have a single row each. Lauren McCarthy and Kyle McDonald's artwork *Vibe Check* is one of the single row works, as it only uses one technology (emotion recognition) and only has one sentiment assigned to it.

When analysing the data you thus need to first select the variables you are interested in, then remove duplicates. To create the chart in Fig. 1 showing the years works were released, we used the following code in R to select the relevant variables, and then removed duplicate rows with the distinct() function. At that point we could safely plot the data without duplicates.

CreativeWorks %>%select(WorkID, Genre, Year) %>%distinct()

In Excel you would do this by deleting columns you do not need, then using the “Remove duplicates” tool on the Data toolbar. It is important to keep the WorkID column, or else removing duplicates will only keep the years and genres, and you will not know how they relate to the number of works.

### 1.3 Situations

Situations provide a second layer of data embedded within a Creative Work. A Situation captures granular details of what humans, technologies, and other agents are doing when machine vision technologies are present in a Creative Work, facilitating analysis of how agency is enacted and distributed between human and non-humans in art, games and narratives. The SituationID is included both in *creativeworks.csv* and *situations.csv,* allowing the files to be combined as desired.

The main unit of analysis is the verbs in each situation, which describe how human and non-human agents in a situation interact with the machine vision technologies. The verbs and agents are in the file *situations.csv*. We have also included *situation_descriptions.csv*, which gives the written descriptions of each situation as entered in the database by the research team, and which also includes short verbal quotes from the text of the work, if relevant. Finally, *situation_visuals.csv* includes free tagged descriptions of the colours and aesthetic qualities that characterise each situation, and a Boolean field indicating whether or not the situation is presented from the point of view of a machine, as when seen “from a drone's perspective” for instance. The colours and aesthetic qualities are not as systematically gathered as the other data in the dataset.

The main file, *situations.csv* includes three data types about each of the 874 identified situations: content description, agents in the situation, and the action taking place. Description data includes the title of the Situation and genre of the Work it is in. We differentiate between three types of agents: characters, entities and technologies. Data depicting agential action in the situation is characterized by verbs, formulated to allow for and distinguish between active and passive actions. Table 6 gives full descriptions of each column in situations.csv.

**Table 6:** Fields in the file situations.csv.

Column header	Description	Multiple values possible
SituationID	A number that uniquely identifies the situation in the dataset.	No
Situation	The situation title includes the title of the work the situation is in followed by a parenthesis with few words describing the situation.	No
Genre	The genre that the Situation belongs to, either Art, Narrative or Game.	No
Character	The name of the character. Characters are agents (i.e. they engage in some kind of activity with the machine vision technology) with at least one identifying trait, e.g. they are *adult humans*, or *bisexual, adult cyborgs*. Additional data about each Character (Species, Age, Ethnicity, Gender, and Sexuality) can be found in c*haracters.csv*.	Yes
CharacterID	A number that uniquely identifies the character in the dataset.	Yes
Entity	A generic agent where details about species, age, ethnicity, gender, sexuality or such details are not relevant or available to us. This field uses a fixed vocabulary: Corporation, Creator, Environment, Government, Humans in general, Image, Law enforcement, Military, Object, User.	Yes
Technology	Machine vision technologies as defined in Table 2.	Yes
Verb	Actions taken by Characters, Entities or Technologies in the Situation. Verbs are an open vocabulary only limited by their form: they either end in *-ing* to indicate a more active stance or end in *-ed* to indicate a more passive or receptive stance. Verbs were assigned with the requirement that they fit in the sentence "This character/entity/technology is ___ing" or "This character/entity/technology is ___ed." Like the other data, these are qualitative interpretations or descriptions of actions in the Situation and are not automatically extracted from the works.	Yes

### 1.4 Characters

The *characters.csv* file lists the 778 Characters that interact with machine vision technologies in the Creative Works. The variables for each character are species, gender, race or ethnicity, age, and sexuality (see Table 7). The ethical considerations taken when making statements about nuanced and sensitive identity traits like race, gender and sexuality, even for fictional characters, are discussed in the Ethics Statement at the end of this paper.

**Table 7:** Fields in characters.csv.

Column header	Description of contents
CharacterID	A number that uniquely identifies this item in the dataset.
Character	The name or title of a Character. If the name can be mistaken for another character in the dataset, it is followed by the title of the Creative Work in which the character appears, in parentheses. Quotation marks are used to indicate personas, or representations of real people. For an explanation of personas see the Ethics Statement below.
Species	Animal, Cyborg, Fictional Species, Human, Machine, Unknown.
Gender	Female, Male, Non-binary or Other, Trans Woman, Unknown.
RaceOrEthnicity	Asian, Black, White, Person of colour, Immigrant, Indigenous, Complex, Unknown.
Age	Child, Young adult, Adult, Elderly, Unknown.
Sexuality	Homosexual, Heterosexual, Bi-sexual, Other, Unknown.
IsGroup	A Boolean true/false variable, where TRUE means that the entry describes a groups of several people acting together.
IsCustomizable	A Boolean true/false variable, where TRUE means that the character can be customized by the user. All customizable characters in the dataset are player-characters in video games.

90 of the characters are “group” characters, such as the Gamemasters in *The Hunger Games*. These characters have the value “TRUE” in the column IsGroup. For group characters we have assigned values to shared traits and marked other traits as Unknown. For example, in *The Hunger Games*, the Gamemasters are all adult and human, but they include both men and women, and while the three most prominent are White, Lucia, who has a minor role in the first movie, is Black. Rather than allow multiple values for a trait we have opted to leave the trait as “Unknown” in cases like this. The Gamemasters’ sexual preferences are not all made explicit, so Sexuality is also marked as “Unknown”. Unknown is thus a term that can include cases where a trait is not made explicit in the work as well as cases where the trait is not applicable to the character or group of characters.

There is one row for each character, unlike *creativeworks.csv* and *situations.csv* where there are multiple rows for each Work or Situation since we allowed multiple values in the same field. There are a few characters where we could have allowed multiple values. The most obvious would be for three characters who are explicitly represented as identifying as transgender women: the robot Paladin in Annalee Newitz's novel *Autonomous* (2017), Max Lao in the game *Technobabylon* (2015), and “Chelsea Manning” as represented in Heather Dewey-Hagborg's artwork *Probably Chelsea* (2017). We could have tagged them as both transgender and as women, but instead chose to use the single category “transgender woman”. There are no explicitly transgender male characters interacting with machine vision technologies in the 500 works we have analysed. There are also cases like the machine animals in *Horizon Zero Dawn* (2017), which could have been given both values for Species: machine and animal. We decided to avoid multiples and choose the most salient trait, so, although we regret the reductionism, the machine animals are simply machines in the dataset.

### 1.5 Interoperablity

The dataset includes Wikidata IDs for each creative work and each creator (artist, author, producer etc.) when available. Wikidata IDs combine the letter Q with a unique numeric data. For instance the game *Horizon Zero Dawn* has the Wikidata ID Q20155528. More data about any item that has a Wikidata ID can be found at a URL ending in the ID, so data linked to *Horizon Zero Dawn* is available at https://www.wikidata.org/wiki/Q20155528.

Wikidata IDs for works are in the file *worksinfo.csv* while IDs for creators are in *creators.csv*. The Wikidata IDs can be used to connect the dataset to other data. Many works, like *Horizon Zero Dawn,* have a lot of data linked through Wikidata, including names of contributors, its budget, the awards it has been nominated for and much more, and its Wikidata page also links to the work's unique ID in other datasets, like on IMDb, Metacritic or even on fandom wikis. Data can be automatically fetched from Wikidata, for instance using the WikidataR package for R or a SPQRL query. The Wikidata ID makes it easier to connect this dataset to other existing or future research datasets about the same works, so long as they include Wikidata IDs.

Unfortunately Wikidata covers mainstream movies and video games well, but contemporary digital art is almost invisible in Wikidata. We could not find other robust ontologies or data sources for digital art, either. Databases of art tend to be connected to individual museums, or in some cases, to national or regional collaborations between museums. For instance, the National Portrait Gallery in the UK and the National Gallery of Canada have databases with information and unique identifiers for artworks in their collections, while Europeana, Calisphere and Digitalt museum, for example, have digitised images from European, Californian and Norwegian archives, art museums and other collections, respectively. There are subject-specific databases, like the Art and Surveillance database (http://www.artandsurveillance.com) or the Rhizome Artbase for born-digital artworks (https://artbase.rhizome.org/wiki), but they do not display unique identifiers that allow for connection to other databases. This means that art that is not in a museum collection or in an auction house database can rarely be connected to any existing datasets. This is the case for most of the artworks in the Machine Vision in Art, Games and Narratives dataset as they are digital and have only been shown online or at festivals and exhibitions.

Most of the games and movies in our dataset have Wikidata IDs, because they are registered on IMDb, which feeds into Wikidata. However, indie games and movies are often not included. Although novels have ISBN numbers and are well-documented in library catalogues, a lot of the novels in our dataset were not in Wikidata, and this was especially the case for novels not published in North America or the UK. Short stories almost never have Wikidata IDs. We chose not to include ISBNs since they would only work for novels, and not for short stories or other kinds of narrative.

## 2 Experimental Design, Materials and Methods

### 2.1 Selection Process

Data was collected between January 2019 and October 2021. The main selection criteria were: that one or more of the 26 identified machine vision technologies (Table 2) was used or represented in the work; that the work could be categorized as a video game, an artwork, or a narrative; and that machine vision was thematized in the work. We interpreted narrative broadly to include novels, movies, electronic literature etc.

Creative Works were selected by using a strategic sampling technique aimed at documenting a wide array of both popular and outlier examples, so as to capture both mainstream representations of machine vision and more experimental approaches.

The selection method combined expert knowledge of the relevant fields of cultural production with systematic searches of existing databases guided by a grounded theory framework of “saturation.” The core team (Rettberg, Kronman, Solberg and Gunderson) have graduate degrees in comparative literature, media art history, gender studies, English literature, digital culture, and have published research on video games, digital art, fan fiction, electronic literature, and narratology. Kronman is also a practicing digital artist with over a decade's experience in the field of digital art. To consolidate our sampling, we searched the databases listed in Table 8 using the names of technologies as keywords. We also used algorithmic recommendations on platforms like Steam and Goodreads, which suggest games and novels similar to ones already viewed, and used Instagram and Twitter to find artists and other works. The data collection team received training in social science classification methods of iteration, coding and intercoder reliability.

**Table 8:** Databases used to find examples of games, digital art and narrative where machine vision technologies are central.

Database	Genre	Information used
Rhizome	Digital Art	Keywords in titles
Archive of Digital Art	Digital Art	Keywords, category search
Ars Electronica Archive	Digital Art	Prix Ars Electronica winners
AI Art Gallery	Digital Art (using machine learning)	Searched collection for works by groups underrepresented in media art and AI
Computer Vision Art Gallery	Digital Art (focus on computer vision)	Searched collection for works by groups underrepresented in media art and AI
Art and Surveillance database	Surveillance art	Searched collection for works by groups underrepresented in media art and AI
Worldcat	Narrative (Novels, short stories)	Titles, book blurbs
Google Books	Narrative (Novels, short stories)	Full text search
Steam	Games	Tags, suggestions
IMDB	Narrative (Movies, TV shows)	Titles, summaries
Archive of Our Own	Narrative (Fan fiction)	Tags, keyword search
Creepypasta Wiki	Narrative (Creepypasta)	Tags, full text search
ELMCIP	Narrative (Electronic literature)	Tags, platforms, descriptions
Goodreads	Narrative (Novels, short stories)	Titles, quotes, similar literature
Dictionary of Surveillance Terms in Science Fiction	Narrative (Science fiction novels and movies)	Surveillance terms

In addition, we considered works nominated or shortlisted for awards and competitions or exhibited at relevant festivals, exhibitions and conferences, which were attended by team members both physically and digitally. A snowball sampling method was employed to find more works by directors, authors and artists we had identified as engaging with machine vision technologies in their works. Saturation was reached quickly for some kinds of technology (e.g., neural networks for image generation), prompting us to stop collecting more examples and move on to other technologies or genres.

Our selection did not aim at data completeness or universality. Given the human-level method of classification, the interpretive qualitative guiding framework, and the vast number of works that reference machine vision, we aimed to instead capture a broad range of examples that could provide material to understand different ways in which machine vision is represented and used in art, narrative and games.

There are artists, particularly those working with artificial neural networks like Mario Klingemann or Memo Akten, whose whole body of work uses machine vision technologies. To include works by a broader scope of artists we decided on a limit of three works per artist. When choosing representative artworks from an artist's or artist collective's body of work, widely exhibited artworks that had been experienced or seen by database authors at exhibitions (also online) were prioritized.

When it comes to fan fiction, searching for stories that featured machine vision in meaningful ways turned out to be more challenging than expected. Even when selecting for fiction based on works that include MV technologies, the available search tools were inapplicable for the task of identifying relevant stories, as the presence of machine vision technologies is not commonly flagged in titles, tags, or blurbs. As a result, the genre is not widely represented in the database.

A considerable effort was made to ensure diversity in our sample of works and creators; however, the dataset is skewed towards English language and Euro-American cultural contexts, as shown in Fig. 2 above. In the case of artworks Dr. phil. Graziele Lautenschlaeger, a Brazilian media artist, curator and researcher in media history and theory was employed. She identified and partly translated 20 works from Latin America to be added into the database. Diana Arce, an Alaskan-born Dominican artist, researcher, and activist based in Berlin, Germany, was consulted to bring forth underrepresented groups in the media arts. We prioritized inclusion of works reflecting perspectives of BIPOC and LGTBQ+ communities, but chose not to collect sensitive personal information about creators’ gender or sexuality. To increase the diversity of narratives, we hired a group of ten students to search for relevant movies and novels-. The group consisted of advanced undergraduate and MA level students in English literature, French literature, Digital Culture and Nordic literature at the University of Bergen; several also had undergraduate training in Media studies and Film studies. Half of the students were immigrants to Norway, providing further cultural and linguistic diversity. They additionally searched for movies, short stories and novels from outside of North America and Western Europe, for example using online lists of sci-fi from specific areas or cultures as well as anthologies and articles about global sci-fi. We provided initial training and group-based and individual follow-ups as the students worked, and the core team doublechecked each of their entries.

**Fig. 3:**
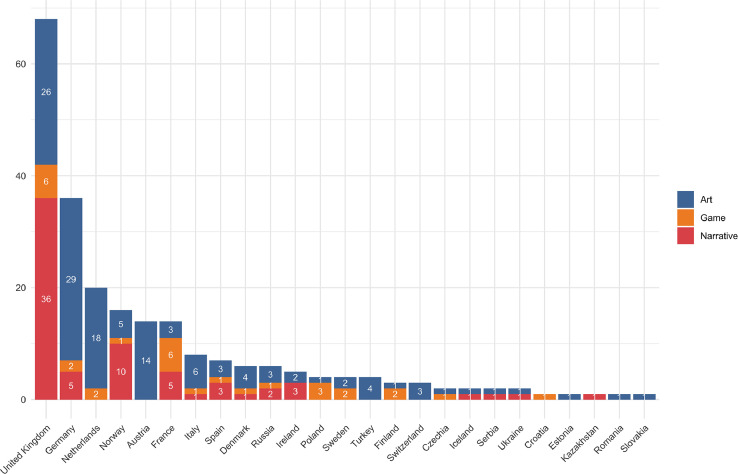
European and Central Asian works in the dataset. Note that some works are affiliated with several countries.

A country-by-country breakdown shows the UK dominating the European data (see Fig. 3), largely because of the dominance of English language narratives. The distribution of artworks is less Anglo-centric than that of narratives. Since artists and other creators are internationally mobile, the country or countries assigned to an artwork can include the creator's country of origin, their country of residence, and the country where the work was produced. This may mean that the overrepresentation of the United States, the United Kingdom and Germany is caused partly by the fact that many artists and production companies are located in the US, UK and Berlin, although the people creating the art, games or narratives may be from other parts of the world.

### 2.2 Developing the Method

The data was entered into a relational database built in Drupal (http://machine-vision.no). The database architecture is an adaptation of the ELMCIP Electronic Literature Knowledge Base [4]. The dataset documented in this paper was exported from the database and includes most of the data fields.

**Fig. 4:**
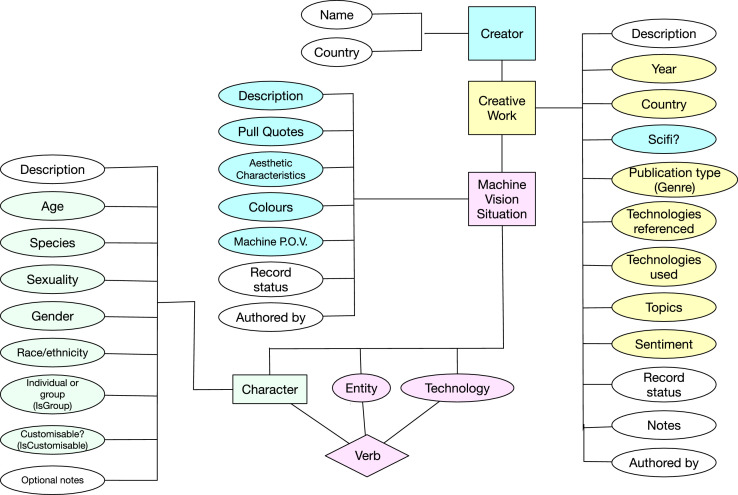
A diagram of all fields in the database showing the relationships between them. Fields included in this dataset are shown with a coloured background. Rectangular boxes indicate content types and oval boxes are variables with a fixed vocabulary.

The data structure was developed iteratively by discussing and interpreting a small initial selection of Creative Works where machine vision technologies were particularly salient. The final organization of the database is shown in Fig. 4. Fields that are included in this dataset have been given coloured backgrounds.

Most fields in the dataset contain interpretative rather than objective data. Standard metadata like the Year of publication and the Title of the Creative Work were usually easy to find but assigning a Topic or a Sentiment required an act of interpretation. Including data that is known to be interpretative rather than assumed to be an objective ground truth is typical of digital humanities projects [5]. We worked to ensure consistency by developing the data structure iteratively as a team and tagging many works together as a group. This collaborative, open-ended classification and annotation process occurred early in the project. Once we reached a point of intercoder reliability in group analysis of qualitatively generated data, where we agreed on most of the tagging, we worked individually.A more detailed explanation of the classification and annotation process vouches for the robustness of our final dataset: Following multiple, collaborative analysis sessions amongst the research team, which involved open-ended, qualitative and thematic analysis of the content, we established a fixed vocabulary to create data labels for the Topics and Sentiments associated with each Creative Work. This analysis was necessarily iterative and adjustable as new Works were annotated, since the team found that early labels did not always fully capture key aspects of the new Works. This common practice in grounded theory coding requires continual assessment, expansion and/or adjustment as new Works are analysed.

When individuals noted low confidence in their own or others’ annotations, intercoder reliability was reassessed: The group would convene to repeat the collaborative and open-ended classification and annotation process noted above, until intercoder realiability was again reached.

We developed the concept of a Machine Vision Situation to capture granular details of what humans, technologies, and other agents are doing in specific interactions with machine vision technologies. We wanted to avoid falling into common binary assumptions [6] about how humans either use technologies as simple tools and remain fully in control (techno-optimism) or how technologies determine culture and leave humans little autonomy (technological determinsm). Our first attempts fell into these traps. For example, we tried a model in which we tagged characteristics and sentiments of the ’operators’ of machine vision technologies as well as the people ‘watched’ through machine vision. An early methodology paper from the project [7] describes this, but also notes how we were experimenting with a more open coding to avoid the binary assumption that a person always operates machine vision as a tool to watch another person. These experiments led to our current data structure. Two of the core team members, Ragnhild Solberg and Marianne Gunderson, came up with the alternative model of assigning an active or passive verb to Characters, Technologies and Entities in a Machine Vision Situation, and we developed and fine-tuned this model as a team.

The resulting classification and annotation process described in this section provides data that is less anthropocentric and deterministic than our first attempts. Rather than always assuming that human actors are the most important or that technologies determine use, our model allows us to see how agency is distributed between multiple agents. The verbs assigned to Characters, Entities and Technologies in a Situation describe different kinds of agency. This enables analyses that explore the *assemblage* of different agents in a situation, for example in a posthumanist or feminist new materialist framework [8], [9], [10], or using situated data analysis [11].

## Ethics Statement

The data was not scraped from websites or existing databases, and consists of standard bibliographic metadata and original, qualitative and interpretative analysis data. The dataset does not contain personal data apart from the names of artists, authors and other creators of published or publicly available creative works, and there is no need to anonymise it.

*Race, gender and sexuality of fictional characters*

The variables for gender, sexuality, and race/ethnicity are used to describe how characters are represented in the work. Bias is a frequently cited problem in machine vision technology [12], [13], [14], and to be able to analyse bias in how machine vision is represented in art, games and narratives it was necessary to collect data about gender, sexuality and race/ethnicity. We approach these labels as socially constructed categories and acknowledge that they may have overlapping and contradictory content and meanings in different contexts. This is especially reflected in the race or ethnicity field, where we have chosen to include multiple labels that reflect different ways in which characters may be seen as racialized or ethnically “other”. Other datasets documenting the gender or race of characters in movies or video games tend to use standard demographic categories from a specific country [15]. This is not possible when collecting data globally as we have done, because race and ethnicity are read differently in different cultural contexts.

Many of the works lack explicit information about characters’ gender, sexuality, or race, so we have relied on discursive and cultural markers to induce the character's characteristics when possible and used the tag “Unknown” when this information is ambiguous or missing.

*Personas: Representations of real people*

Some of the Characters we have registered are representations of real people, such as when a performer appears in a music video. In these cases, the data collected describe the persona the person is portraying in this particular work, and do not necessarily correspond to the actual person's characteristics.

*Ethical assessment of using data about creators*

The Norwegian Centre for Research Data (NSD) found that this dataset is in compliance with the GDPR (reference code 833684). The project processes general categories of personal data, but only minimally (names of creators, countries creators live in or are affiliated with). The project processes personal data on the legal basis that processing is necessary for the performance of a task carried out in the public interest, cf. the General Data Protection Regulation art. 6 nr. 1 e), cf. art. 6 nr. 3 b), cf. the Personal Data Act § 8. The assessment of NSD is that the processing meets the requirement of scientific research, cf. the Personal Data Act § 8, and therefore constitutes a task in the public interest.

For each Creative Work, we register the creator name and their country of origin/residency, but we have not collected any additional information about the creators of the works.

Some of the creative works are informally published and their authors may not have the same expectation of publicity as when a work is formally published or exhibited. In these cases, the Creator field only documents the name that the creator has published under, which is commonly a pseudonym. For these entries, information about country of origin is not included, as this is usually not known. All data collected from openly published original fiction published on online platforms is in compliance with each of the platforms’ redistribution policies.

## CRediT authorship contribution statement

**Jill Walker Rettberg:** Conceptualization, Methodology, Investigation, Data curation, Writing – original draft, Writing – review & editing, Visualization, Supervision, Project administration, Funding acquisition. **Linda Kronman:** Methodology, Investigation, Data curation, Writing – original draft, Writing – review & editing. **Ragnhild Solberg:** Methodology, Investigation, Data curation, Writing – original draft, Writing – review & editing. **Marianne Gunderson:** Methodology, Investigation, Data curation, Writing – original draft, Writing – review & editing. **Stein Magne Bjørklund:** Software, Data curation. **Linn Heidi Stokkedal:** Methodology, Investigation, Data curation, Project administration. **Kurdin Jacob:** Investigation, Data curation, Project administration. **Gabriele de Seta:** Formal analysis, Writing – review & editing, Project administration. **Annette Markham:** Methodology, Writing – review & editing.

## Declaration of Competing Interest

Linda Kronman is a member of the artist collective KairUs, which has three works of art included in the dataset. The authors declare that they have no other known competing financial interests or personal relationships which have or could be perceived to have influenced the work reported in this article.

## Data Availability

A Dataset Documenting Representations of Machine Vision Technologies in Artworks, Games and Narratives (Original data) (Dataverse).
